# Prevalence of mental disorders, associated co-morbidities, health care knowledge and service utilization in Rwanda – towards a blueprint for promoting mental health care services in low- and middle-income countries?

**DOI:** 10.1186/s12889-022-14165-x

**Published:** 2022-10-05

**Authors:** Yvonne Kayiteshonga, Vincent Sezibera, Lambert Mugabo, Jean Damascène Iyamuremye

**Affiliations:** 1 Mental Health Division, Rwanda Biomedical Center, Kigali, Rwanda; 2 Department of Clinical Psychology, Center for Mental Health, College of Medicine and Health Sciences, University of Rwanda, Huye, Rwanda; 3 Center for Mental Health, University of Rwanda, Kigali, Rwanda

**Keywords:** Rwanda, Mental health, Genocide survivors, Healthcare knowledge, Mental health service utilization, Mental illness, Low- and middle-income countries (LMIC)

## Abstract

**Background:**

In order to respond to the dearth of mental health data in Rwanda where large-scale prevalence studies were not existing, Rwanda Mental Health Survey was conducted to measure the prevalence of mental disorders, associated co-morbidities and knowledge and utilization of mental health services nationwide within Rwanda.

**Methods:**

This cross-sectional study was conducted between July and August 2018, among the general population, including survivors of the 1994 Genocide against the Tutsi. Participants (14–65 years) completed the Mini-International Neuropsychiatric Interview (Version 7.0.2), sociodemographic and epilepsy-related questionnaires. General population participants were selected first by random sampling of 240 clusters, followed by systematic sampling of 30 households per cluster. Genocide survivors within each cluster were identified using the 2007–2008 Genocide Survivors Census.

**Results:**

Of 19,110 general survey participants, most were female (*n* = 11,233; 58.8%). Mental disorders were more prevalent among women (23.2%) than men (16.6%) (*p* < 0.05). The most prevalent mental disorders were major depressive episode (12.0%), panic disorder (8.1%) and post-traumatic stress disorder (PTSD) (3.6%). Overall, 61.7% had awareness of mental health services while only 5.3% reported to have used existing services. Of the 1271 genocide survivors interviewed, 74.7% (*n* = 949) were female; prevalence of any mental disorder was 53.3% for women and 48.8% for men. Most prevalent disorders were major depressive episode (35.0%), PTSD (27.9%) and panic disorder (26.8%). Among genocide survivors, 76.2% were aware of availability of mental health services, with 14.1% reported having used mental health services.

**Conclusions:**

Despite high prevalence of mental disorders among the general population and genocide survivors, utilization of available mental health services was low. A comprehensive approach to mental health is needed for prevention of mental illness and to promote mental healthcare services.

**Supplementary Information:**

The online version contains supplementary material available at 10.1186/s12889-022-14165-x.

## Background

Globally, approximately 450 million people suffer from mental and behavioral disorders, with approximately one person in four developing such a disorder during their lifetime [1]. Worldwide, the burden of mental illnesses poses serious public health challenges, with approximately 7.4% of the global burden of disease attributed to mental disorders [1, 2]. In 2010, mental and substance use disorders were the fifth leading cause of disability-adjusted life years (183.9 million) [2]. In 2015, 17.9 million years were lost to disability due to mental disorders in Africa; a 52% increase from 2000 [3].

### National context

Rwanda, an East African country with a population of approximately 12 million, has come a long way since 1994 when the country experienced the devastating genocide against the Tutsi. Over 100 days, more than one million people were killed and survivors were subjected to extreme levels of physical and psychological violence [4]. Health systems were destroyed, and the traumatic events of the genocide gave rise to a high prevalence of mental health problems [5]. During the genocide period, 37% of men and 35% of women experienced at least one traumatic event such as rape, witnessing an unnatural death or forcibly made to flee their home [5]. In addition, multiple studies conducted at various time points after the genocide highlight a number of mental illnesses, such as post-traumatic stress disorder (PTSD), depressive disorders, and substance misuse disorders [6–9]. Further, in an evaluation of mental healthcare in post-genocide Rwanda, the most frequently diagnosed disorders in the adult population were psychotic disorders, substance use disorders, depression, and epilepsy [4].

The Rwanda government through its health sector placed mental health among its priorities; by ensuring the accessibility of mental health services, hiring and training of medical professionals, purchasing medicines, and raising awareness about mental health.

Rwanda’s Ministry of Health has expanded access to health services, including mental health. The organization of a mental healthcare program has helped the communities to embark on a trauma healing process and to create conditions where social cohesion and productive economic participation can be restored.

To reduce mental health morbidity and improve service accessibility within the community, the Rwandan Ministry of Health introduced (1995) and later (2011) revised a mental health policy [10] whose focus was the integration and decentralization of mental health services within the primary healthcare.

The government of Rwanda has decentralized and integrated mental health services from national referral hospitals down to health centers, where trained health professionals (including psychiatrists, mental health nurses, clinical psychologists, and general nurses and GPs) conduct assessment of, and provide care and treatment for, a wide range of mental health needs. This includes mental health units at district hospitals, which provide mostly individualized psychotherapy and pharmacotherapy to diagnosed patients. However, the scale of need for mental health support far outstrips current capacity.

In 2018, a government-mandated nationwide study (Rwanda Mental Health Survey; RMHS) was conducted to investigate the prevalence of mental disorders in the general population, and particularly, among genocide survivors living in Rwanda. While some epidemiological surveys conducted in countries such as Nigeria and South Africa have provided useful data about the prevalence of mental disorders, large-scale community studies are very rare in Africa [11].

The overall objectives of this study were to estimate the prevalence of common mental disorders and identify associated risk factors. Additionally, the study aimed to assess the level of knowledge and utilization rates of conventional and unconventional mental health services within the general population of Rwanda. Meeting the study objectives would provide evidence needed to develop policies and strategies to promote mental health care services.

## Methods

### Overall population study

#### Study design

**Fig. 1:**
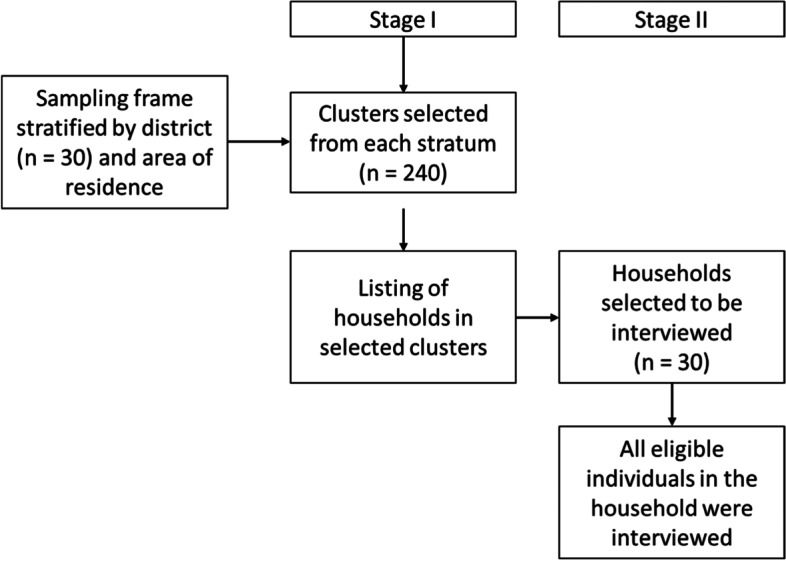
Sampling procedure used to identify study population

The RMHS was a cross-sectional study that integrated two distinctive population samples, the general population and genocide survivors in Rwanda. The sampling procedure used to identify the study population is presented in Fig. 1. The sample size for this study was calculated using a 95% confidence interval and a 5% margin of error, to arrive at a sample size of 6750 households. This sample size was further increased to 6888 households to account for a predicted 2% non-response rate. Sample size calculations are further detailed in Additional file 1.

#### Eligibility criteria

The general population sample was computed at the district level with a sampling frame based on geographical clusters (enumeration areas) defined previously in the 2012 Rwanda Population and Housing Census [12]. Eligible participants were Rwandan citizens aged 14–65 years old who were residing in Rwanda at the time of the survey. Participants were required to have been resident in their respective enumeration area for at least 6 months. For the general population survey, the minimum age to participate was 14 years old. Individuals with a limited capacity of communication that prevented the interviewer from verbally completing the surveys were excluded.

#### Data collection and analysis

The main survey instrument for this study was the Mini-International Neuropsychiatric Interview, version 7.0.2, for the Diagnostic and Statistical Manual of Mental Disorders, 5^th^ Edition (DSM-5) [13]. This instrument has been used before in the context of Rwanda [14, 15]. To distinguish between symptoms of mental disorders and epilepsy, participants were asked additional questions on their medical history using a survey designed for this study (see Additional file 1). Data on demographic variables such as sex, age, marital status, educational level, employment, and economic status (as measured by Ubudehe categories, reflecting their degree of social and economic vulnerabilities [see Additional file 1]) [16], were collected. In addition, data on the utilization of mental healthcare services, including community health workers, religious and traditional healers, and reasons for not seeking mental health support were also collected.

#### Prevalence time frames

In order to identify the prevalence of previous or current mental disorders, the Mini-International Neuropsychiatric Interview applied different time frames to the individual mental health disorders. Participants were diagnosed with major depressive episode based on current, past or recurrent episodes, with an episode characterized by the presence of symptoms that persist for at least two weeks. The MINI instrument classifies suicidal behavior disorder as current in participants with symptoms present in the previous 12 months or in remission for those whose symptoms occurred in the previous 1 to 2 years. No time limits were applied to diagnostic criteria for bipolar disorder, major depressive disorder with psychotic features, or any psychotic disorder; participants were assessed on the presence of any current or previous symptoms within their lifetime. Panic disorder was diagnosed based on current (past month) or lifetime occurrence. Prevalence of alcohol or substance use disorders was evaluated over the preceding one-year period. Participants were diagnosed with social phobia, obsessive–compulsive disorder (OCD) or PTSD if diagnosis criteria was met in the past month. Assessment of antisocial personality disorder was by lifetime prevalence.

#### Study conduct

Overall, 79 individuals were recruited and trained as field staff members, including 60 data collectors, 15 team leaders and four supervisors and data collection was carried out August 1–31, 2018. The RMHS protocol was reviewed and approved by the Rwanda National Ethics Committee (RNEC) (Ref: 0061/RNEC/2018 dated February 15, 2018) and the National Institute of Statistics of Rwanda prior to data collection. Written and oral informed consent, in the participant’s native language (Kinyarwanda), was obtained from the participants in the study.

### Genocide survivors’ subset study

#### Study design

The subset study was conducted in the same way as the overall study. The sample size for the subpopulation of genocide survivors was calculated using a 95% confidence interval with a 2.5% margin of error, resulting in a sample size of 900 individuals; this was amended to 918 to allow for the predicted 2% non-response rate.

#### Eligibility

To be a survivor of the genocide, an individual should have been in Rwanda no later than December 30, 1994, so the minimum age to participate was 24 years old. The genocide survivors’ survey sample was computed at national level using information from the 2007‒2008 Genocide Survivors Census, which was obtained from the National Institute of Statistics of Rwanda.

#### Data collection and analysis

Before starting data collection activities, a pilot survey was conducted to get the enumerators acquainted with the study procedures including listing process, how to approach the household, obtaining participant consent, ensuring confidentiality of the study participants, and to evaluate study instruments. When the main survey started, participants were included in the survey if they fulfilled the inclusion criteria including being a Rwandan citizen residing in Rwanda, aged 14 to 65 years old, and having lived in the enumeration area for at least 6 months. Males and females with limited capacity of communication that prevent the interviewer from oral administration of the surveys were excluded from the study. Genocide survivors who took part in the general population survey were excluded from participating in the study for the genocide survivors.

Data analysis was directed by the flow of the Mini-International Neuropsychiatric Interview instrument. Firstly, dichotomous variables were computed combining a series of questions for each module of the Mini-International Neuropsychiatric Interview in order to be able to estimate the prevalence of mental disorders, whereby a ‘Yes’ meant that criteria for a disorder was met and a ‘No’ meant the opposite. Descriptive statistics were calculated for all variable characteristics. In order to understand the relationships between the prevalence of mental disorders and population characteristics, a chi-square test for independence was used. Weighting procedures were performed for the general population survey, in order to allow for extrapolation of results to the target population. Data analyses were performed using STATA 15.

### Role of the funding source

The funders of the study had no role in study design, data collection, data analysis, data interpretation, or writing of the report. The corresponding author had full access to all data in the study and had final responsibility for the decision to submit the manuscript for publication.

## Results

### Population demographics

Overall, 19,110 respondents participated in the general population survey where female participants represented 58. 8% (*n* = 11,233). Participation varied by age group of the respondent with those aged 26–35 being the most represented with 25.3% of the sample followed by 36–45 age group with 19.4%. The oldest group, aged 56–65, was the least represented with 10.6%. Majority of respondents (56.5%) had completed primary school, followed by 25.9% who were either illiterate or had not completed primary school. The respondents who attended university represented only 2.2%. Among the respondents, 32.1% had never married, 40.2% were married, 3.7% were separated or divorced, 7.1% were widowed and 16.8% reported to be living together as if married. Of the total respondents, 52.3% of them reported being self-employed, 43% were unemployed, 4.1% were salaried employees and 0.6% were underage of labor force (Table 1).

**Table 1:** Demographic characteristics of survey participants

Frequency, n (%)	General population (*N* = 19,110)	Genocide survivors (*N* = 1271)
**Sex**		
Male	7877 (41.2)	322 (25.3)
Female	11,233 (58.8)	949 (74.7)
**Age group, years (g*eneral population)***		
14–18	2825 (14.8)	
19–25	3358 (17.6)	
26ؘ–35	4830 (25.3)	
36–45	3711 (19.4)	
46–55	2354 (12.3)	
56–65	2032 (10.6)	
**Age group, years (genocide survivors subpopulation)**		
24ؘ–34	275 (21.6)	
35–44	314 (24.7)	
45–54	309 (24.3)	
55–65	373 (29.3)	
**Residence**		
Urban	2669 (14.0)	NE
Rural	16,311 (85.4)	NE
**Marital status**		
Never married	6143 (32.1)	139 (10.9)
Married	7683 (40.2)	692 (54.4)
Living together as if married	3209 (16.8)	238 (18.7)
Divorced/separated	715 (3.7)	61 (4.8)
Widowed	1360 (7.1)	141 (11.1)
**Education**		
Illiterate and primary school not completed	4958 (25.9)	310 (24.4)
Primary school	10,802 (56.5)	664 (52.2)
Secondary/technical and vocational education and training	2934 (15.4)	224 (17.6)
University	416 (2.2)	73 (5.7)
**Ubudehe category**		
Category 1	2842 (14.9)	297 (23.4)
Category 2	7996 (41.8)	520 (40.9)
Category 3	8160 (42.7)	452 (35.6)
Category 4	9 (0.0)	1 (0.1)
Unknown	103 (0.5)	1 (0.1)
**Employment**		
Salaried employee	634 (3.3)	76 (6.0)
Self-employed	8973 (47.0)	796 (62.6)
Unemployed	4350 (22.8)	399 (31.4)
Under age of labor force	5153 (27.0)	-

*NE* Not evaluated

### Prevalence of mental disorders

**Fig. 2:**
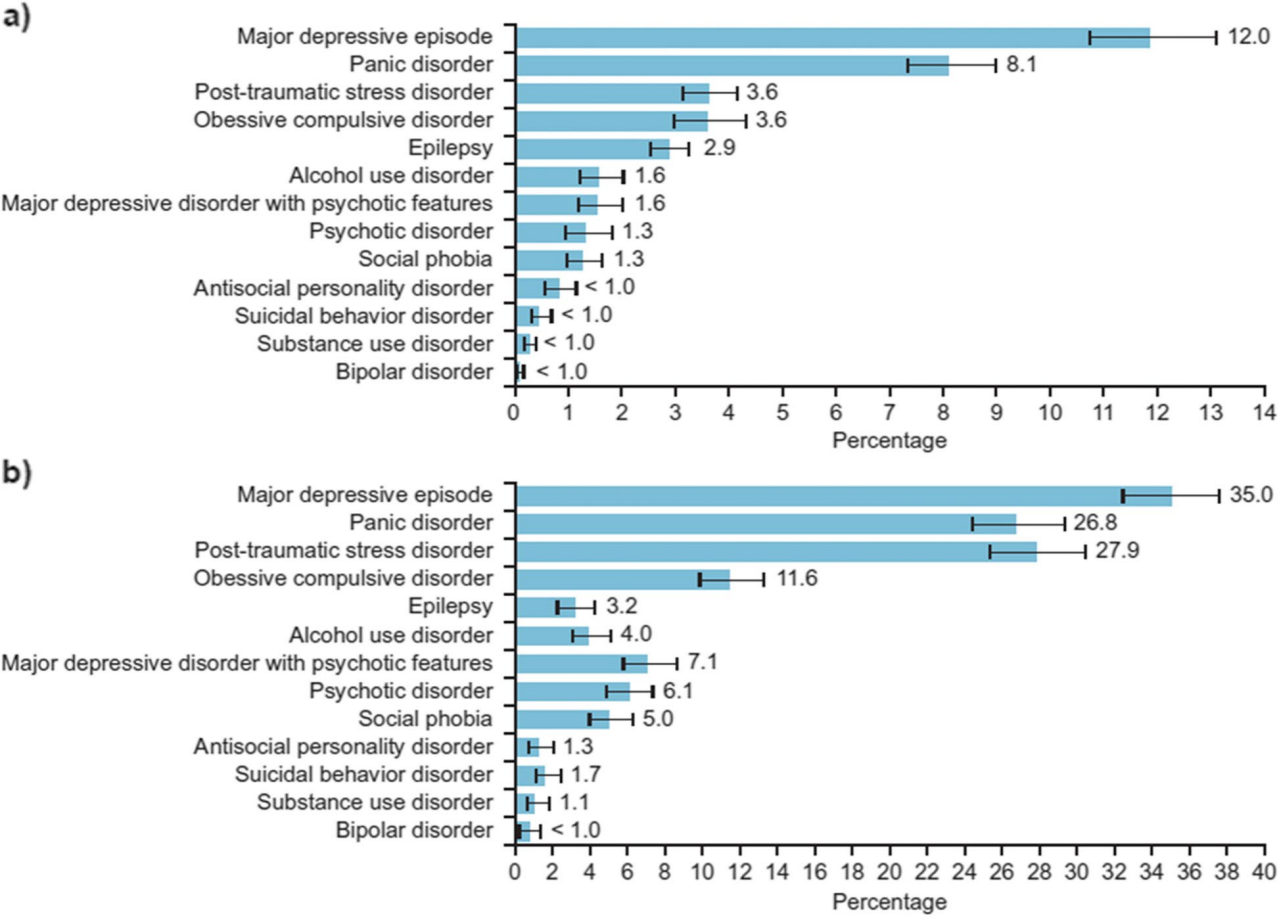
Prevalence of mental disorders among (**a**) the general population (*N* = 19,110) and (**b**) genocide survivors (*N* = 1271)

Overall, the prevalence of one or more mental disorders among the general population was 20.49% (*n* = 3915). Of the 19,110 participants surveyed, major depressive episode was the most prevalent mental disorder, with 12.0% of the population meeting the diagnostic criteria, followed by panic disorder (8.1%) (Fig. 2a). Post-traumatic stress disorder and OCD showed similar prevalence rates of 3.6%, followed by epilepsy (2.9%). Psychotic disorders and social phobia were identified in 1.3% of respondents. There were similar prevalence rates of major depressive disorder with psychotic features and alcohol use disorder (1.6%) (Fig. 2a). The least reported mental disorders (< 1%) were antisocial personality disorder, suicidal behavior disorder, substance use disorder, and bipolar disorder (Fig. 2a).

#### Associated co-morbidities

**Fig. 3:**
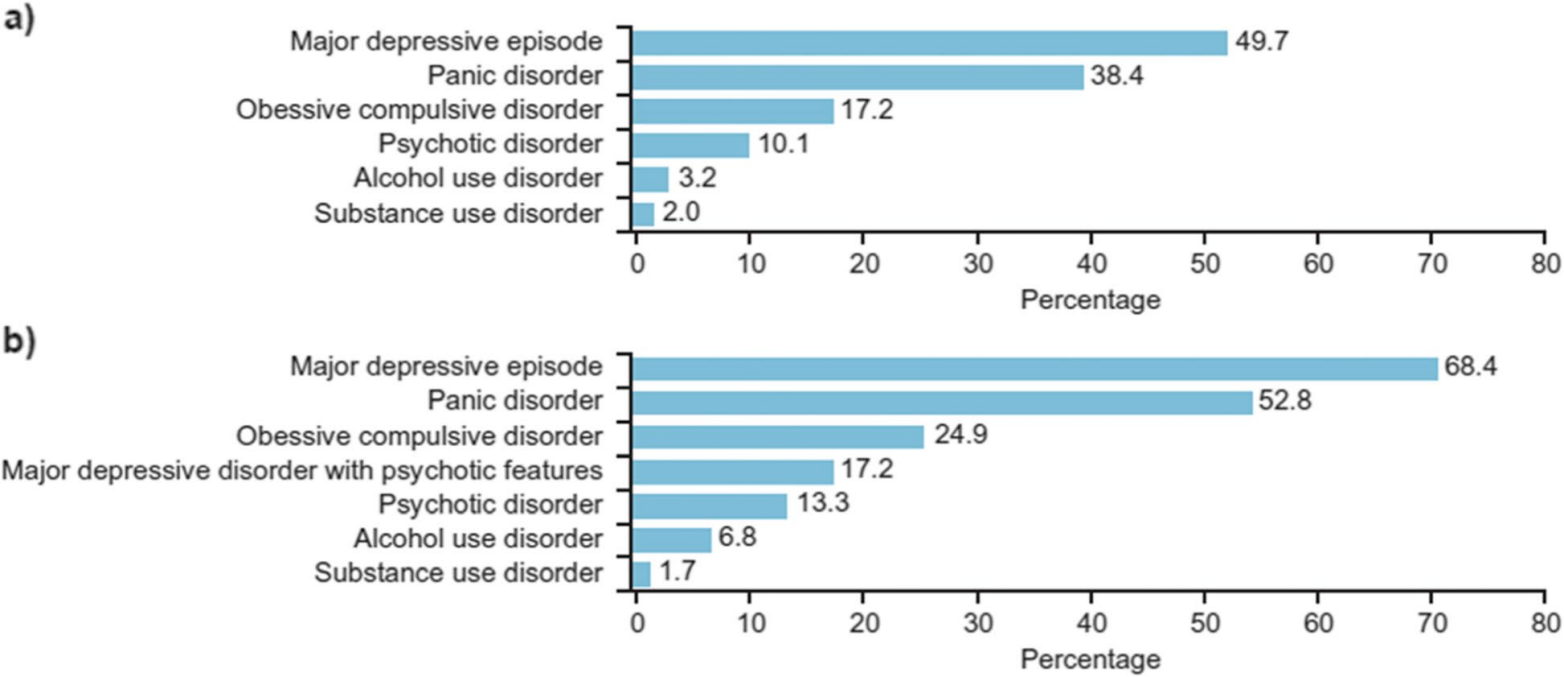
Co-morbidity of PTSD with major mental disorders among (**a**) the general population (*N* = 19,110) and (**b**) genocide survivors (*N* = 1271)

Of the general population participants who met the criteria for PTSD, 49.7% were also affected by major depressive episode and 38.4% met the criteria for panic disorder. Another 17.2% of participants who met the criteria for PTSD had co-morbidity with OCD, while 10.2% of those with PTSD also met the criteria for psychotic disorders. Only 2% of those who met the criteria for PTSD had substance use as co-morbid disorder (Fig. 3a).

### Socio-demographic differences in mental disorders

Overall, more women were affected by mental disorders than men, where the prevalence of one or more mental disorders was 23.2% vs. 16.6%, respectively (*p* < 0.05). Further, more women were affected by major depressive episode than men (14.4% [95% CI: 15.1–13.8] vs. 8.2% [8.9–7.7]; *p* < 0.05). For women, the prevalence of PTSD was also significantly increased compared with men (4.4% [95% CI: 4.8–4.0] vs. 2.6% [3.0–2.3]; *p* < 0.05), as was panic disorder (10.2% [9.6–10.8] vs. 5.2% [4.7–5.7]; *p* < 0.05) and OCD (4.2% [3.9–4.6] vs. 2.7% [2.4–3.1]. Additionally, women showed a greater than two-fold higher prevalence of suicide behavior disorder (0.7 [95% CI: 0.5–0.8]) than men (0.3 [0.2–0.4]) (*p* < 0.05). In contrast, men had a significantly increased prevalence of alcohol and substance use disorder compared with women; 3.4% (95% CI: 3.1–3.9) of men met the criteria for alcohol use disorder compared with 0.3% (0.2–0.5) of women (*p* < 0.05) and substance use disorder was twice as prevalent in men (0.4 [0.3–0.6]) compared with women (0.2 [0.1–0.3]) (*p* < 0.05).

Overall, an increase in age was associated with a higher risk of being affected by mental disorders, although there was a drop in prevalence among those aged 56–65 years old. Major depressive episodes were the most prevalent at older ages, being most common in 46–55 year olds (18.0% [19.6–16.5]). Participants aged 14–18 years had the lowest prevalence of major depressive episode (4.0% [4.8–3.4]). An exception to the above trend was the prevalence of antisocial personality disorder, which was less common as age increased; prevalence was greatest (1.1%) among 14–18 (95% CI: 0.7–1.5) and 19–25 year olds (0.8–1.5) while prevalence was lowest (0.3%) among 56–65 year olds (0.1–0.6). In addition, the prevalence of suicidal behavior disorder and OCD was greatest among 26–35 year olds (0.8% [95% CI: 0.5–1.0] and 4.5% 3.9–5.0], respectively).

The prevalence of mental disorders was inversely associated with the educational achievement of the population; those who achieved higher education had the lowest prevalence of mental disorders (12.5%), whereas prevalence was greatest among participants considered illiterate and those who did not complete primary school (24.1%) (*p* < 0.05).

In urban settings, the prevalence of mental disorders was slightly higher (21.3%) compared with rural settings (20.3%), although the difference was not statistically significant (*p* > 0.05). By Ubudehe categorization, mental disorders were most prevalent among participants classed as Category 1 and Category 2, except for alcohol use disorder and psychotic disorder, which were most prevalent in participants classed as Category 3 (3.7% [95% CI: 0.0–21.7] and 12.2% [1.1–38.1], respectively). Many mental disorders such as major depressive episode, were more prevalent among those who were divorced/separated or widowed (28.1% [95% CI: 31.4–25.0] and 28.1% [30.5–25.7], respectively), compared with those who were never married or married (7.1% [7.7–6.5] and 11.0% [11.7–10.3], respectively), although no statistically significant differences were observed.

### Awareness of mental health services

Among the general population, 61.7% were aware of where they could seek support for mental health. For the general population who knew where to find mental health support, healthcare facilities were the most common mental health service identified (90.1%), followed by community health workers (38.8%). Traditional and religious healers were also recognized as options for mental health services (Fig. 3).

### Mental health service utilization

Reported utilization of mental health services for the general population stands at 5.3%. Among those who reported utilization of mental health services, just over three quarters utilized healthcare facility services, followed by 32.8% who used services provided by religious healers. Approximately 29% of participants sought services from traditional healers while only around one in four were served by community health workers.

In most individuals who met the criteria of having mental disorders there was very little utilization of available resources. Just 25.0% of respondents who met the criteria of psychotic disorder reported to utilize mental health support and only 11.5% of those meeting the criteria of PTSD reported utilization of mental health support. The proportion was even lower for other mental disorders, whereby only 6.1% of those meeting the criteria for alcohol use disorder reported utilization of mental health support.

### Reasons for not utilizing mental health services

Among participants who met the criteria for one or more mental disorders, the most common reason given for not seeking mental health support was that the individual did not know that mental health is a problem that required medical treatment (40.5%; *n* = 722). Other reasons given for not seeking support were lack of money (39.6%; *n* = 277), unable to get to location of services (32.5%; *n* = 117) and fear of being stigmatized (27.1%; *n* = 108).

### Genocide survivors’ subset

#### Participant demographics

Reflecting the timing of the genocide (1994), most genocide survivor survey respondents were aged 55–65 years (29.3%), while 24–34 year olds were the least represented age group (21.6%) (Table 1). Of the 1271 genocide survivors interviewed throughout all 30 districts of Rwanda, 74.7% (*n* = 949) were female. Demographic characteristics of the respondents are summarized in Table 1. Most genocide survivors interviewed were from the Southern province (39.4%); just 6.9% of participants lived in the Northern Province.

#### Prevalence of mental disorders

Among the genocide survivors, 52.2% (*n* = 663) were identified as having one or more mental disorders. Similar to the general population survey, major depressive episode was the most prevalent mental disorder among genocide survivors, accounting for 35.0% of disorders identified (Fig. 2b). Just over a quarter of genocide survivors were identified as having PTSD and panic disorders (27.9% and 26.8%, respectively), while 11.6% met the criteria for OCD (Fig. 2b). The prevalence of epilepsy was 3.2%. As with the general population survey participants, the least prevalent (< 2%) mental disorders among genocide survivors were suicidal behavior disorder, antisocial personality disorder, substance use disorder, and bipolar disorder (Fig. 2b).

#### Associated co-morbidities

Among the genocide survivors who met the criteria for PTSD, 68.4% of participants also met the criteria for major depressive episodes. Another 52.8% of participants meeting the criteria for PTSD also met the criteria for panic disorder. Co-morbidity of PTSD with OCD was 24.9% while co-morbidity with substance use disorder was 1.7% (Fig. 3b).

### Socio-demographic differences in mental disorders

As with the general population, more women were affected by major depressive episodes than men (36.0% [33.0–39.1] vs. 32.0% [27.1–37.2), respectively). A trend was observed between major depressive episode and age group of the participants; those aged 55–65 years had the highest prevalence (44.8% [39.8–49.8]) while the younger group (24–34 years) had the lowest prevalence (22.5% [17.9–27.8]). Throughout all four provinces and City of Kigali, major depressive episode was most prevalent in the City of Kigali (40.1% [32.2–48.5]).

The level of education achieved was associated with the prevalence of mental disorders; among participants who were illiterate and did not complete primary school, the incidence of major depressive episode was greatest (44.5% [39.1–50.1]). Major depressive disorder was least common in those who attended university (13.7% [7.3–22.8]). As with other disorders, prevalence of PTSD and panic disorders was greater among individuals with lower education levels (33.5% and 28.4%, respectively).

#### Comparisons with overall population in Rwanda

Compared with the general population survey participants, the prevalence of PTSD was markedly higher among genocide survivors (3.6% vs. 27.9%, respectively) and was equally common among male and female survivors (28.3% and 27.7%, respectively). Similar to the general population, among genocide survivors who met the criteria for PTSD, the most common co-morbid conditions were major depressive disorder, panic disorder and OCD. Panic disorders were more prevalent among genocide survivors (26.8%) compared with the general population (8.1%); genocide survivors aged 45–54 years presented the highest prevalence (30.4%) whereas among the general population, prevalence was greatest in those aged 56–65 years old (11.5%). In both the general population and among genocide survivors, prevalence was greatest among those who were illiterate and did not complete primary school (10.0% and 28.4%, respectively). As with the general population, the least prevalent mental disorders were suicidal behavior disorder (1.7%), antisocial personality disorder (1.3%), substance use disorder (1.1%) and bipolar disorder (0.7%).

#### Awareness of mental health services

**Fig. 4:**
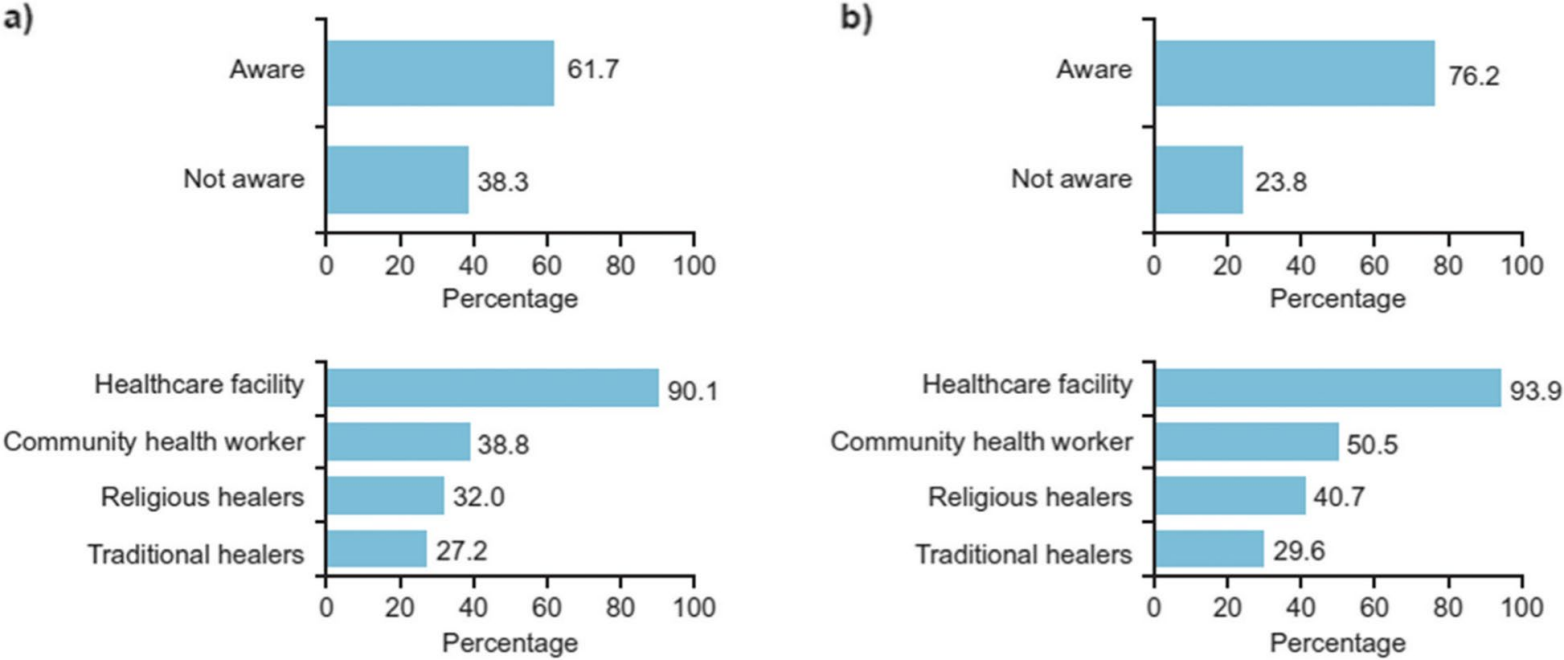
Awareness of mental health services (**a**) among general population (*N* = 19,110) and (**b**) genocide survivors (*N* = 1271)

Of the 1271 genocide survivors, 76.2% were aware of the availability of mental health services. Similar levels of knowledge of where to find mental health support were observed with the genocide survivors; health facilities were the most known level of mental health services (93.9%). Compared with the general population, a greater proportion of genocide survivors were aware of mental health support being available from community health workers (50.5%). Genocide survivors also recognized traditional and religious healers as options for mental health services (Fig. 4).

### Mental health service utilization

For genocide survivors, the mental health services utilization rate was 14.1%; 85.9% of survivors reported they did not use mental health services. Of the services available to genocide survivors seeking support, healthcare facilities were the most utilized service (12.9%), followed by community health workers (3.9%). Survivors also sought traditional and religious healers for support (2.2% and 2.7%, respectively).

## Discussion

### Applicability of study

Given the dearth of mental health information especially in low-income countries, large-scale epidemiological studies such as the RMHS are also useful to the global community, whose understanding of mental health issues in LMICs is restricted due to lack of data [17]. To our knowledge this is the largest survey related to mental health in a low- and middle-income country. Gathered evidence reflected the overall population, with a broad range of characteristics, such as geography, age, sex, education level, and marital and economic status. Clearly this study could be adapted for other countries where understanding of mental health issues is lacking.

Data relating to a subset of individuals victims of the genocide were also captured. However, interpretation of the comparisons with the overall population should be made with caution, given that the current study was not intended to draw such comparisons.

### Prevalence of overall mental disorders

The overall prevalence of mental health disorders in this study in Rwanda (approximately 20% of the population) was higher than that previously reported in the overall population of Rwanda in 2017, which was 12.72% of the population [18]. Richie and Roser highlighted that the data sources in LMICs have limited raw data and under-reporting. We therefore consider the prevalence reported here to be a closer reflection of the true value in Rwanda. Data from smaller studies that were collected in Rwanda broadly support our findings, but is worth noting that the variability was extensive [6–9]. Furthermore, Richie and Roser report relatively consistent prevalence of data of mental health within Africa (typically between 10.0% and 17.5%). This prevalence was lower than reported in high income countries (United States 17.34% and Canada 15.51%, respectively) [18]. As noted above, mental health disorders remain widely under-reported, particularly in LMICs, where data are scarce [18]. Our findings could therefore suggest (due to the reasons that Richie and Roser outlined), that previous data available in LMICs may be an underestimation.

Our data are in closer agreement with an earlier systematic review of global period and lifetime prevalence of common mental disorders and less recent WHO World Mental Health Survey Initiatives [19–21]. In the systematic review analysis, the overall period (12-month) or lifetime prevalence of patients in LMICs who had experienced a mental disorder ranged from 17.6%–29.2% [19]. In the WHO World Mental Health Survey Initiative the 12-month prevalence was estimated at 9.8%–19.1%; the global range of lifetime prevalence of mental health disorders was more variable (12.0%–47.4%; 18.1%–36.1%) [20, 21]. However, the only data collected in Africa in the WHO World Mental Health Survey Initiative were in Nigeria and South Africa (global lifetime prevalences: 12.0% and 30.3%, respectively) [20, 21], again suggesting significant variability. Given that South Africa and Nigeria were the only two African countries which participated in the World Mental Health surveys, our findings show further research into the prevalence of mental disorders in LMICs is required.

Overall, major depressive disorder, panic disorder, PTSD, and OCD were the most common mental disorders diagnosed in the general population. The current findings illustrate that prevalence of common mental disorders in Rwanda are consistent with other published data regarding the global prevalence of mental disorders. Previous studies conducted in Rwanda reported high prevalence of mental disorders including PTSD, depression, and anxiety [6–9]. Studies into PTSD found prevalence ranging from 11% [9] to 46% [8], while depression prevalence was found between 15% [6] and 53% [7].

### Socio-demographic differences in mental disorders

In our study females and those with lower level of education had increased prevalence of mental disorders. This is consistent with the 2010 Global Burden of Disease study [2], where women also had a greater burden of mental disorders than men; this finding is consistent across both high income and LMICs [2, 22]. Secondly, low educational attainment has previously been shown to be associated with increased prevalence of mental disorders [23].

### Awareness of mental health services

Despite the high prevalence of mental disorders, awareness of mental health services was low. It has previously been documented that there are challenges when it comes to engaging mental health service users in LMICs such as Nigeria and Uganda; a lack of service user participation and low mental health literacy were barriers identified across six LMICs [24].

### Mental health service utilization and barriers to utilization

In spite of the high prevalence of mental disorders among Rwandans, utilization of mental health services remains very low. Many Rwandans who have mental disorder do not recognize it as a serious health issue that requires treatment. This is due, in part, to poor cultural acceptance of mental disorders as normal or ignorance surrounding mental disorders. In some LMICs, such as Nigeria, some mental disorders are viewed as karma, or divine or universal punishment for previous sins [25]. Such cultural beliefs can lead to discrimination against people with mental illness, preventing them from seeking treatment [26]. In addition, while the majority of participants sought treatment from health facilities, some participants still resort to traditional healers or religious leaders; societal beliefs in traditional healers and prayers has previously been documented in other African countries, such as Kenya and Uganda [27].

During the study, some challenges were reported as a hindrance to mental health service utilization, such as fear of being stigmatized or difficulties to travel to where services are provided. The association between stigma and the utilization of mental health services has been documented previously in Rwanda [27]; research has also highlighted the detrimental impact of stigma on the utilization of mental health services throughout Europe [28]. Additionally, people with mental disorders who live far from a centralized treatment facility—the majority of the population in most LMICs—are often unable to access care, and as such, those with common mental disorders, who collectively account for more than half of the total global mental health burden worldwide, are most often left untreated [2, 29].

### Genocide survivors’ subset

A stark difference was observed in the prevalence of mental disorders between the general population and genocide survivors, with major depression and panic disorder among genocide survivors being threefold higher compared with the general population. This finding is unsurprising given the horrific events they experienced, as well as the long-term effects suffered by survivors and their families [4].

### Addressing the burden of mental health

Despite the significant increase in years lost to disability as a result of mental and substance use disorders, research which defines the prevalence and associated response to mental health disorders is lacking, particularly in LMICs [3, 27]. The present study not only provides information about mental health to inform decision makers about required programs and activities, but also serves as an inspiration for future research into mental health, which will support the understanding of mental health in general, and particularly in LMICs. As such, the present study has multiple implications, especially at the national level but also at the international level.

### Increasing mental health service utilization

Over the last 15 years, the Rwandan government has invested heavily in delivery of quality health care. As such, the Rwandan Health Sector Policy 2015 identified mental health as a priority area of intervention; this recommends the integration of mental health services into all national health system structures, including at the community level [16]. Hence, mental healthcare has been decentralized in general hospitals and integrated into primary healthcare in order to increase the accessibility and quality of mental health services [4].

While many steps have been taken towards improving mental healthcare within Rwanda, certain challenges remain. In order to promote the utilization of mental health services, the general public should be made increasingly aware of services available. Government entities should develop strategies for, and lead on the creation of mobile health clinics which will play an important role in bringing in-person and on-line services closer to populations in need, along with online services that allow remote provision of mental health service delivery. Examples of successful models include the Friendship Bench [30]. Recommendations for how mental health structures at all levels of the health system (including building infrastructure, increasing awareness and patient support) could be improved are presented in Table 2.

**Table 2:** Summary of recommendations for the overall population and more specific recommendations around victims of Genocide

Population	Level	Recommendations
Overall	Infrastructure building	• Take a comprehensive approach to mental health from promotion through prevention ◦ treatment, long-term care of, and recovery from mental disorders including concurrent substance misuse disorders • Create specific programs/packages to strengthen mental health services at different levels of health system • Reinforce the capacity of health personnel especially at the community level to be able to assess and treat early symptoms of mental disorders • Strive to ensure that mental health resources address unmet needs of the population ◦ support effective mental health promotion, early intervention, prevention, service provision and programs
	Increasing awareness	• Promote the need for equality for mental health and recognition of the links between mental and physical health in achieving health and wellbeing • A high proportion of individuals do not seek mental health support; thus it is important to develop programs to raise the awareness as well as utilization of mental health services • Promote access to and quality of evidence-based primary, secondary and specialist mental health care, which is integrated across health and social care to meet the needs of the citizens • Strengthen coordinated efforts to develop better and sustainable community mental health systems such as community forums that provide support to community members with mental problems
	Patient support	• Challenge stigma and discrimination experienced by people with mental disorders through approaches that are based on evidence and respect for human rights • Recognize that individuals with mental disorders are at high risk of abuse, violence, detention or hospitalization, and promote the least restrictive mental health interventions, treatment, and care wherever possible
Genocide victims	Patient support	• Take action to address the social determinants of mental health, in a cross-sectoral and holistic manner in order to reduce both physical and mental health disparities so that Genocide survivors have an equal chance to lead healthy lives • Increase the focus on improving the mental health and wellbeing of Genocide survivors to improve mental health outcomes for this and future generations • Increase public health actions that promote resilience, reduce risks, and address the root causes of chronic stress that threaten family and caregiving environments in order to protect and promote mental health of Genocide survivors

Considering the Ministry of Health’s strategic plan for mental health, the present findings could contribute to the concerted efforts for improving mental health in the Rwandan population and provide a platform upon which to build future research into mental health. Stigma and associated discrimination against people suffering from mental disorders is an important obstacle to overcome and will require a multilayered approach to increase public knowledge around mental health, change negative attitudes towards mentally ill individuals [31, 32] and give voice to people suffering from mental disorders to reduce stigma [33].

### Strengths and limitations of the study

One of the key strengths of the current study was the strong methodology utilized, namely stratified random sampling and applied sampling weights for each estimate of mental disorder, which strengthen the accuracy of estimates provided throughout the report. Additionally, as a large-scale prevalence study, data collection for RMHS covered the entire country thus increasing the degree of precision of estimates provided by the survey. However, the present study has some limitations. As a cross-sectional study, information on risk factors as well as prevalence was collected at the same time. This hinders the understanding of temporal relationship between mental disorders and risk factors, therefore cautious interpretation of the relationship is needed. Since the surveys were conducted in households, the RMHS potentially missed out on individuals with serious mental illness who may have not been present in the home at the time of the survey, thereby under-counting prevalence.

Further, prevalence rates of common mental disorders in Africa vary greatly depending on the research instrument used and the population sampled [34]. Finally, the current study evaluated mental disorders over differing time periods, did not allow for fair comparison of overall prevalence rates. However, the current findings benefit from data collection using the Mini-International Neuropsychiatric Interview, the reliability and validity of which have been previously documented [35].

## Conclusions

Despite the high prevalence of various mental disorders in both the general population and genocide survivors, significant proportions of the population are either not aware of the availability of mental health support or do not use the services available. Our findings highlight the need to improve the availability, accessibility and quality of mental healthcare at all levels in order to improve mental healthcare for individuals affected by mental disorders.

## Supplementary Information

**Additional file 1.**

## Data Availability

The datasets used during the current study are on file within the Rwanda Biomedical Center. Data are available upon reasonable request and with permission of the Director General of the Rwanda Biomedical Center.

## Abbreviations

CI: Confidence interval
GP: General practitioner
LMIC: Low- and middle-income countries
NE: Not evaluated
OCD: Obsessive–compulsive disorder
PTSD: Post-traumatic stress disorder
RNEC: Rwanda National Ethics Committee
RMHS: Rwanda Mental Health Survey
