# Supplementary material for: Prevalence of mental disorders, associated co-morbidities, health care knowledge and service utilization in Rwanda – towards a blueprint for promoting mental health care services in low- and middle-income countries?
Source: BMC Public Health. 2022 Oct 5;22:1858. doi: 10.1186/s12889-022-14165-x (PMC9533613; doi:10.1186/s12889-022-14165-x)
Supplement: Supplementary file 1 — Additional file 1. [file 12889_2022_14165_MOESM1_ESM.docx]

**Supplementary information**

**Methods**

*Sample size*

For the general population and genocide survivor population, sample sizes were calculated slightly differently. The sample for general population survey was computed taking into account the domains which are the 30 districts of Rwanda, whilst the sample for genocide survivors’ survey was calculated at the national level; in other words the domain being national level. The following formula was used to compute both samples:

n = Deff 2 (1/𝑃−1)2

Where

n = sample size

Deff = design effect (1.5)

P = assumed prevalence (0.5 recommended when prevalence is not known)

e = margin error

For the present study we used 95% confidence interval (Z = 1.96) and the latter was used to compute the margin error. For the general population sample a recommended value of 0.05 was used to compute the sample size, and for the sample of genocide survivors’ survey the recommended margin error value of 0.025 was used to compute the sample size.

Calculation of the sample size for the general population survey gave an estimated minimum sample size of 225 households per domain. For the sample for the genocide survivors’ survey, calculation procedures provided an estimated total sample size of 900 individuals.

Given the total number of 30 domains and the sample size calculation estimated 225 households per domain, the total sample size required for the general population survey was 6750 (n = 225*30). Sample sizes were adjusted for the non-response effect; a non-response of 2% was assumed and, consequently, the estimated sample size for the general population survey became 6888 (6750/0.98), whereas the estimated sample size for the genocide survivors’ survey became
918 (900/0.98).

For the general population survey, sampling procedures were carried out in two stages: the first stage involved sampling of the enumeration area stratified by district and area of residence and were selected with probability proportional to size. An enumeration area is a village or part of a village created for 2012 Rwanda Population and Housing Consensus; these enumeration areas served as the primary sampling unity for the Rwanda Mental Health Survey.

The first stage provided 240 clusters, eight clusters per district selected independently with probability proportional to the enumeration area’s measure of size. In the selected enumeration areas, a listing procedure was performed such that all households were listed. This procedure was important for correcting errors existing in the sampling frame, and it provided a sampling frame for household selection. The second stage involved equal probability systematic sampling of 30 households within each of selected enumeration areas. A specific form was used during listing to identify eligible households.

For the genocide survivors’ survey, the process began with the identifying the number of genocide survivors available in each district using the data from 2007‒2008 Genocide Survivors Census provided by the National Institute of Statistics of Rwanda. After identifying the number of genocide survivors in a district, the sample size was dispatched proportionally to the number of genocide survivors living in the District.

*Data collection*

‘Ubudehe’ refers to the categorization of residents reflecting their degree of social and economic vulnerabilities: Families who do not own a house and can hardly afford basic needs (Category 1); those who have a dwelling of their own or are able to rent one but rarely get full time work (Category 2); people who are employed or are employers (including those with small and medium enterprises who can provide employment to dozens of people) (Category 3); big business owners, people working in international organizations or industries and public servants (Category 4) (Government of Rwanda, 2015).

*Epilepsy-related questionnaire*

Participants were asked questions (shown below; translated from Kinyarwanda language) relating to their medical history in order to distinguish between symptoms of mental disorders and epilepsy.

| - Have you ever had an illness which caused you to fall down unconscious? |
| --- |
| - When you fall down do you blank out? |
| - Have you ever bit your tongue as a consequence of falling down? |
| - Have you found yourself dirty after falling down? |
| - Have you ever get injured as a consequence of falling down? |
| - Most commonly when you blank out how long does it last? |
| - Do you know what cause you to blank out? |
| - How old were you when you experienced your first ever epileptic seizure? |
| - How many epileptic seizures did you have during the first year of your epileptic attacks? |
| - How many epileptic attacks have you had in the past year? |
| - How long has it been since your last epileptic seizure? |
| - Is there any family member of yours who experienced similar symptoms? |
| - Have you had prescribed medications by a recognized physician? |
| - Before you experience those symptoms:   - Have you been diagnosed with severe malaria or meningitis?   - Have you had a head injury which resulted in loss of consciousness? |
